# Preparation of dissociated mouse primary neuronal cultures from long-term cryopreserved brain tissue

**DOI:** 10.1016/j.jneumeth.2019.108452

**Published:** 2020-01-15

**Authors:** M. Cano-Jaimez, E. Tagliatti, P.R.F. Mendonca, E. Nicholson, U. Vivekananda, D.M. Kullmann, K.E. Volynski

**Affiliations:** Dept. Clinical and Experimental Epilepsy, Queen Square, Institute of Neurology, University College London, London UK

**Keywords:** AP, Action Potential, BSA, bovine serum albumin, DAPI, 4′,6-diamidino-2-phenylindole, DIC, differential interference contrast, DIV, days *in vitro*, DMSO, dimethyl sulfoxide, FBS, foetal bovine serum, mEPCS, miniature excitatory post-synaptic currents, PBS, phosphate buffered saline, PLL, poly-L-lysine, *pv*, average probability of vesicular release, SEM, standard error of the mean, RRP, readily-releasable pool, SV, synaptic vesicle, Cryopreservation, Primary neuronal cultures, Hippocampi, Long-term storage, Synaptic transmission, Action potential

## Abstract

**Background:**

Dissociated primary neuronal cultures are widely used as a model system to investigate the cellular and molecular properties of diverse neuronal populations and mechanisms of action potential generation and synaptic transmission. Typically, rodent primary neuronal cultures are obtained from freshly-dissociated embryonic or postnatal brain tissue, which often requires intense animal husbandry. This can strain resources when working with genetically modified mice.

**New method:**

Here we describe an experimental protocol for frozen storage of mouse hippocampi, which allows fully functional dissociated primary neuronal cultures to be prepared from cryopreserved tissue.

**Results:**

We show that thawed hippocampal neurons have functional properties similar to those of freshly dissociated neurons, including neuronal morphology, excitability, action potential waveform and synaptic neurotransmitter release, even after cryopreservation for several years.

**Comparison to the existing methods:**

In contrast to the existing methods, the protocol described here allows for efficient long-term storage of samples, allowing researchers to perform functional experiments on neuronal cultures from brain tissue collected in other laboratories.

**Conclusions:**

We anticipate that this method will facilitate collaborations among laboratories based at distant locations and will thus optimise the use of genetically modified mouse models, in line with the 3Rs (Replacement, Reduction and Refinement) recommended for scientific use of animals in research.

## Highlights

• Primary neuronal cultures from cryopreserved tissue retain functional neuronal properties.
• Cryopreservation of brain tissue allows long-distance collaborations.
• Exchange of cryopreserved tissue can reduce the need to maintain multiple colonies of mutant mice.

## 1 Introduction

Neuronal networks are generally embedded within complex three-dimensional structures consisting of cell bodies and processes belonging to multiple neurons and glia. This elaborate organisation often imposes technical challenges to dissect, manipulate and analyse phenomena occurring at the level of single synapses. Therefore, dissociated primary neuronal cultures offer several advantages. For example, neurons are less densely packed, allowing perfusion of drugs, transfection with plasmids, and efficient fluorescence imaging of neuronal function. For this reason, primary cell culture techniques have been successfully employed to investigate a variety of cellular mechanisms including neuronal development, passive and active neuronal electrical properties, and mechanisms of synaptic transmission and plasticity.

At present, the most widely used techniques for preparation of rodent hippocampal and cortical neuronal cultures rely on enzymatic dissociation of freshly dissected neuronal tissue from either embryos (Kaech and Banker, 2006) or new-born pups (Beaudoin et al., 2012). Postnatal neuronal cultures present several advantages in comparison to embryonal cultures, including fewer animals required to obtain a given number of neurons. On the other hand, culturing of postnatal neurons has proven to be more challenging, mainly because they are more susceptible to damage during dissection and dissociation (Beaudoin et al., 2012). Both neuronal preparations typically yield neurons with a physiologically relevant proportion of neurons of different types, with a majority of excitatory glutamatergic cells (80%) and the remainder mainly consisting of GABAergic inhibitory neurons (Benson et al., 1994), although less is known of how different subtypes of interneurons are represented.

Primary neuronal cultures have a constrained life-time and a restricted time window for reproducible experiments, with most studies using cells within 14–25 days *in vitro* (DIV). This means that large numbers of animals (often superfluous) are used to prepare neuronal cultures on a regular basis. Therefore, optimising preparation and cryopreservation of freshly dissociated neurons (Otto et al., 2003; Pischedda et al., 2018; Quasthoff et al., 2015) or of brain tissue blocks (which could be used in the future for culturing) (Kawamoto and Barrett, 1986; Negishi et al., 2002a, b; Rahman et al., 2010) is constantly sought.

Here we describe a simple protocol for long-term cryopreservation of postnatal mouse hippocampi and preparation of functional primary neuronal cultures from frozen tissue. We demonstrate that dissociated cultures can be prepared from tissue that has been stored in liquid nitrogen for at least two years. We further show that neuronal cultures produced from cryopreserved hippocampi exhibit morphological and physiological properties similar to those of freshly dissociated neurons.

The developed protocol can be routinely adopted to archive limited tissue from transgenic animals, thus allowing replication of experiments from the same source at different times. Moreover, we anticipate that the use of this method will facilitate collaborations among laboratories based in different locations, and will also reduce the number of animals needed for a specific project.

## 2 Materials and methods

### 2.1 Cryopreservation of hippocampi for neuronal culture

Animal care and use protocols were approved by the UK Home Office. Fresh hippocampi were isolated from individual P0–P1 C57BL/6 J mouse pups under a stereomicroscope and transferred to a 1.5 ml cryovial containing 1 ml of Wash Buffer (Hanks Balanced Salt Solution (Sigma, H9394) supplemented with 5 mM HEPES (Sigma, H4034)) which was immediately replaced by 1 ml of Freezing Solution (10% DMSO and 90% FBS). The gradual freezing step was performed using a Mr Frosty Freezing container, designed to achieve a rate of cooling close to -1 °C/minute (catalogue number: 5100-0001, Thermo Fisher Scientific). Critically, the dissected hippocampi were transferred to the container, which was pre-equilibrated at −20 °C and then placed at −80 °C freezer overnight. The cryo-vials were transferred into the liquid nitrogen tank next day for long-term storage.

### 2.2 Preparation of primary neuronal cultures from cryopreserved or fresh hippocampi

A cryovial with frozen hippocampi from a single mouse pup was removed from liquid nitrogen and quickly thawed in a 37 °C water bath. The contents were kept in suspension by tapping the cryovial side. Once fully defrosted (∼ 3–4 min), the contents of the cryovial were flipped into a 60 mm culture dish containing 10 ml of Wash Buffer. The de-frosted hippocampi were rinsed twice with Wash Buffer to remove traces of DMSO and debris. The hippocampi were then subjected to an enzymatic digestion for 4 min at 37 °C in 1 ml of Incubation Buffer (in mM 122.4 NaCl, 5.0 KCl, 7 Na_2_HPO_4_ and 25 HEPES) supplemented with 0.5% Trypsin (Thermo Fisher Scientific, 15090046) and DNAse I 75 u/μl (D5025-150 KU, Sigma). The same solution was used for digestion of freshly dissected hippocampi when preparing control sister cultures, except that the incubation time at 37^0^C was increased from 4 min to 10 min. The rest of the procedures were performed at ambient temperature (∼21–25 °C). Enzymatic digestion was terminated by addition of 2 ml of Neutralisation Buffer (Wash Buffer supplemented with 10% FBS, Thermo Fisher Scientific, 10082147). The hippocampi were then rinsed twice with Wash Buffer and triturated using a standard p1000 micropipette until most of the tissue was disrupted (up to 10 times). After gravity sedimentation of non-disrupted material (∼ 2 min) the cell suspension was transferred into a new tube, and cells were pelleted by centrifugation at 800 rpm (100 x *g*) for 5 min. The cell pellet was re-suspended in 1 ml of Complete Neuronal Medium (Neurobasal A medium containing 1.2 % Glutamax and 0.1 % B27 supplement (Thermo Fisher Scientific, 10888022). Live cells from either cryopreserved or for freshly dissociated hippocampi were counted using a haemocytometer and trypan blue exclusion assay, and plated onto PLL-coated 19 mm glass coverslips in 12 well plates at ∼ 240,000 cells/well. The typical yield from cryopreserved hippocampi was approximately ∼1.5–2-fold lower than from freshly dissociated tissue (see Results).

### 2.3 Immunocytochemistry

Hippocampal neurons were fixed with 4% paraformaldehyde in PBS for 10 min, permeabilised with 0.2% Triton X-100 in PBS for 30 min, and non-specific binding sites were blocked by incubation for 30 min with 3% BSA in PBS before an overnight incubation at 4 °C with primary antibodies. Neurons were washed 3 times with PBS and incubated with Alexa Fluor-conjugated 488, 568 or 555 secondary antibodies for 45 min at room temperature. Coverslips were washed again 3 times in PBS and mounted on slides using a mounting medium containing DAPI (4′,6-diamidino-2-phenylindole) (Thermo Fisher Scientific, P36931) to visualise nuclei. The following primary antibodies were used: anti-GFAP (G3893, Sigma); anti-Gad67 (MAB5406, Chemicon); anti-Map2 (ab5392, Abcam); anti-Bassoon (SAP7F407, Novus Bioscience); anti-Homer1 (160 023, Synaptic Systems). Control samples without primary antibody incubation gave no signal. Co-localisation between pre and post-synaptic staining was estimated by measuring the overlap coefficient (Szymkiewicz-Simpson coefficient, see JaCoP plugin, Image J), defined as the size of the intersection divided by the smaller of the sizes of the two sets (Li et al., 2010).

### 2.4 Electrophysiological recordings

Passive and active membrane properties of cultured neurons were measured in the whole-cell current clamp configuration. Data were acquired with a CV-7B headstage and a Multiclamp 700B amplifier (Molecular Devices). Custom written National Instruments LabView software was used to control a National Instruments board to generate stimuli and sample recordings at 20 kHz (4 kHz low pass filtered). Briefly, neuronal cultures at 15–20 DIV were placed in a recording chamber of an upright microscope (Scientifica) or of an inverted microscope (Olympus IX71), and perfused at room temperature with Extracellular Recording Solution consisting of (in mM): 125 NaCl, 2.5 KCl, 2 CaCl_2_, 2 MgCl_2_, 30 Glucose and 25 HEPES (pH 7.3 balanced with NaOH). Network activity was reduced with NBQX 10 μM, AP5 50 μM and picrotoxin 50 μM. Neurons were patched-clamped with fire-polished borosilicate glass pipettes (4–6 MΩ, Harvard Apparatus), filled with (in mM): 130 K^+^ Gluconate, 10 KCl, 1 EGTA, 4 ATP-Mg, 0.5 GTP-Na_3_ and 10 phosphocreatine Na_2_ (pH 7.3 balanced with KOH, −10 mV liquid junction potential).

Passive membrane properties were obtained by applying a 500 ms step of hyperpolarising current (−50 to −30 pA), and the initial part of the voltage response was least-square fitted to an exponential curve: Vmt=R×I×e-tτ+Vfinal (where $V_{m}$ is the membrane potential, R is the membrane input resistance, I is the current injected, τ is the membrane time constant, and *V_fina_*_*l*_ is the steady-state membrane potential after current injection).

Firing properties were assessed by measuring action potentials (APs) in response to depolarising 500 ms current pulses (range 10–140 pA). The first AP near the current threshold was used to estimate spike take-off voltage (maximum of the second derivative of membrane potential), spike half-width (when membrane potential reaches the average between take-off and peak) and spike height (difference between the take-off and the peak). All voltages were corrected for the liquid junction potential (−10 mV).

### 2.5 Lentivirus production and neuronal transduction

The production of vesicular stomatitis virus-pseudotyped second-generation pFU_SypHy lentivirus was performed by co-transfection of the expression vectors (pFU_SypHy) and two helper plasmids (pCMVdelta and vesicular stomatitis virus G protein-expressing plasmid) in human embryonic kidney 293 T (HEK293 T) cells using Lipofectamine 2000 (Thermo Fisher Scientific) following the manufacturer’s instructions. The pFU_SypHy plasmid was kindly provided by Dr. A. Maximov (The Scripps Research Institute, La Jolla, USA). Viral titres ranged from 3.8 to 7.5 × 10^8^ transforming units/ml. Primary hippocampal neurons were transduced at 7 DIV at 10x multiplicity of infection. After 21 h, half of the medium was replaced with fresh medium. All experiments were performed between 15 and 18 DIV.

### 2.6 Imaging of vesicular release

SyPhy imaging was performed on an inverted Zeiss Axiovert 200 fluorescence microscope using a 63× (1.4 NA) oil-immersion objective. 12-bit images were acquired throughout the experiment using a Prime 95B CMOS camera (Photometrics) coupled to a 488 nm excitation LED light source and a green 510 long-pass emission filter, with an exposure time of 50 ms. SypHy responses were recorded in an ∼ 180 × 180-μm region of interest (ROI) typically containing 50–200 individual boutons. Primary hippocampal neurons were perfused in Extracellular Recording Solution, supplemented with NBQX (10 μM, Ascent Scientific) and DL-AP5 (50 μM, Ascent Scientific) to prevent recurrent activity, through a laminar flow perfusion system. APs were evoked by field stimulation *via* platinum bath electrodes separated by 1 cm (12.5–15 V, 1 ms pulses). After 1 s of acquisition of baseline fluorescence (F_0_), neurons were stimulated with a single AP or a train of 20 APs at 100 Hz. Single AP stimulation was repeated 10 times with an inter-trial interval of 20 s. Images were analysed using ImageJ (Schneider et al., 2012) and MATLAB software. Synaptic boutons were identified by subtracting the baseline fluorescence from the peak of fluorescence response after 20 AP 100 Hz stimulation. This was followed by measuring the average fluorescence response at all identified boutons in each experiment. After subtracting the background, the data were normalised to the resting pHluorin signal. Six to eight independent cell culture preparations were used in each experimental condition.

### 2.7 Statistical analysis

The distribution of data in each set of experiments was firs*t*-tested for normality using the Shapiro-Wilk test. Normally distributed data are presented as mean ± S.E.M., each plot also contains the individual data points. Student’s *t*-test for group means was used to test for significance. Data sets that failed the normality test are presented using box-and-whisker plots (box 25th–75th percentiles, whiskers 10th – 90th percentiles), each plot also contains the individual data points. Here the Mann-Whitney U test was used as indicated.

## 3 Results

### 3.1 Optimisation of the enzymatic dissociation step

**Fig. 1:**
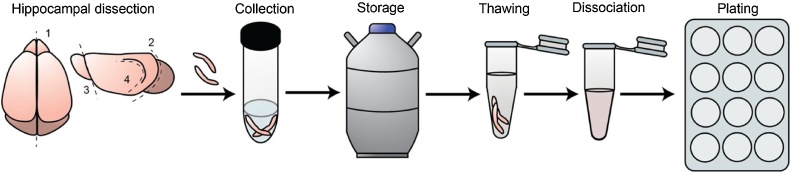
Schematic description of the protocol for preparation of neuronal cultures from cryopreserved hippocampi. Fresh hippocampi from murine P0 pups were dissected, gradually frozen in a freezing solution containing 10 % 10 % DMSO/FBS and stored for up to several years in liquid nitrogen. To prepare the cultures a cryovial containing hippocampi from one animal was quickly thawed in 37 °C water bath and processed for enzymatic digestion and dissociation. Neurons were then plated on PLL-coated coverslips.

**Fig. 2:**
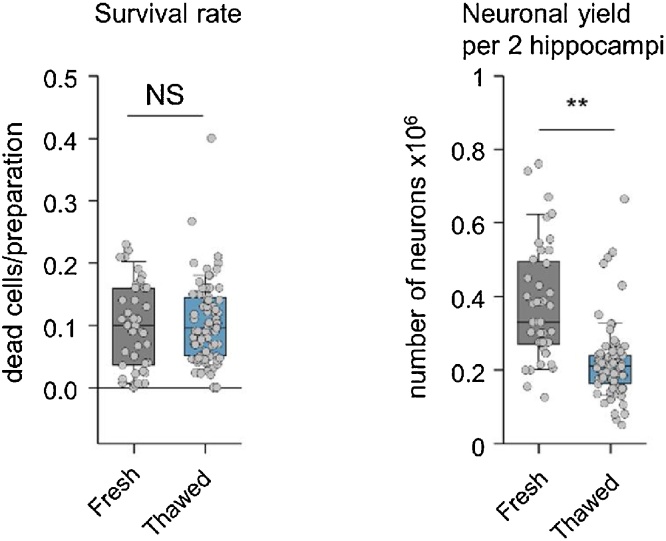
Comparison of neuronal survival and total neuronal yield between cultures prepared from cryopreserved and freshly dissociated hippocampi. Reduction of duration of the enzymatic digestion step from 10 min (fresh hippocampi) to 4 min (thawed hippocampi) resulted in similar neuronal survival rates in both preparations (left, trypan blue exclusion assay), but led to approximately a 2-fold reduction in total neuronal yield. Fresh n = 42, thawed n = 74 preparations. NS p > 0.75, ** p < 0.01, Mann–Whitney U test.

The protocol used to cryopreserve mouse hippocampi and to prepare primary dissociated cultures from the thawed tissue is shown schematically in Fig.1 (see also Methods). Enzymatic dissociation of brain tissue is a critical step in preparation of primary neuronal cultures. Normally, neurons from freshly dissected cortices or hippocampi are dissociated by incubation in 0.25% trypsin for 10–20 min at 37 °C (Beaudoin et al., 2012; Benson et al., 1994; Ermolyuk et al., 2012; Kaech and Banker, 2006). Such treatments are designed to obtain an optimal ratio between the survival rate and the total neuronal yield. We found however, that cryopreservation makes neurons more sensitive to the enzymatic digestion, and 10 min incubation of thawed hippocampi in 0.25% trypsin resulted in death of the majority of neurons (> 50–70 %). We therefore reduced the incubation time to 4 min. This drastically improved the survival rate (∼ 90%), which became similar to that in the freshly dissociated neuronal preparation (Fig. 2a). The reduction of the trypsinisation time nevertheless resulted in approximately a 2-fold decrease in the total yield of neurons obtained from cryopreserved tissue (Fig.2b).

### 3.2 Immunocytochemical characterization of thawed hippocampal cultures

**Fig. 3:**
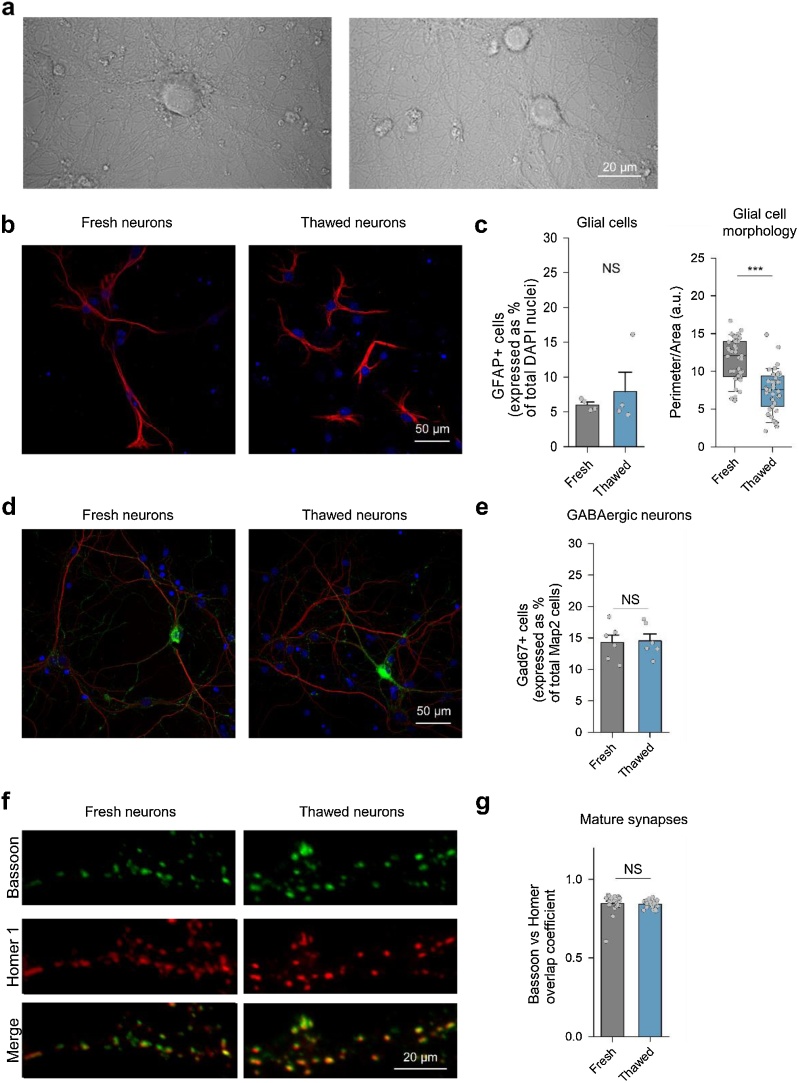
Morphological and immunocytochemical characterisation of thawed and fresh hippocampal cultures. (**a**) Representative differential interference contrast (DIC) images of fresh (left) and thawed (right) neurons at 15 DIV. (**b**, **c**) Thawed and freshly dissociated cultures contain similar proportions of glial and neuronal cells (**b**) Representative images of fresh and thawed cultures stained with anti-GFAP (red) antibody to identify glial cells and DAPI (blue) to stain nuclei of all cells. (**c**) Quantification analysis showing the percentage of GFAP positive glial cells over the total number of DAPI nuclei (left) and the perimeter/area ratio (right), used as a crude measure of the morphological astrocyte complexity (Hasel et al., 2017). Data are means ± SEM, n = 4 preparations. NS p > 0.8, Student’s t-test, *** p < 0.001, Mann Whitney *U* test (**d**, **e**) Thawed and freshly dissociated cultures contain the same proportions of inhibitory and excitatory neurons. (**d**) Representative images of fresh and thawed neuronal cultures stained with anti-Gad67 (green) and anti-Map2 (red) antibodies. (**e**) Quantification analysis showing the fraction of Gad67 positive neurons in the population of Map2 positive cells. Data are means ± SEM, n = 6 preparations. NS p > 0.8, Student’s t-test. (**f**, **g**) Synaptogenesis is similar in thawed and freshly dissociated cultures. (**f**) Representative images of dendritic fragments in fresh and thawed cultures stained with antibodies against Bassoon (green, presynaptic) and Homer 1 (red, postsynaptic) antibodies. (**g**) Quantification of the overlap coefficient between the two markers. Data are means ± SEM, n = 20 from coverslips for freshly dissociated cultures and n = 19 coverslips for thawed cultures from 4 independent preparations, NS p > 0.5 Student’s t-test.

To characterise neuronal cultures prepared from cryopreserved hippocampi, we systematically compared their functional properties to those of cultures prepared from fresh tissue. After 15 DIV both thawed and fresh neurons plated on PLL-coated coverslips formed extensive neuronal networks (Fig. 3a). Both thawed and fresh cultures contained comparable percentage of glial cells, although astrocytes in thawed cultures showed less elongated morphology (Fig.3b-c). To compare the relative proportions of inhibitory and excitatory neurons we double-stained both preparations for the dendritic protein Map2 to reveal all neurons, and for the Gad67 enzyme to identify inhibitory neurons (Fig.3d). Quantification analysis revealed that thawed and fresh cultures contained the same proportions of inhibitory neurons (∼15%) (Fig.3e). We next tested whether cryopreservation had any effect on synaptogenesis. We double-stained thawed and fresh neurons after synapse maturation (15 DIV) (Mozhayeva et al., 2002) with the pre-synaptic marker Bassoon and the post-synaptic marker Homer 1. The overlap coefficients between the pre- and postsynaptic markers were similar in both preparations (Fig. 3f, g). Together, these results argue that thawed neurons undergo maturation comparable to that of fresh neurons.

### 3.3 Electrophysiological characterisation of neuronal excitability and passive membrane properties of neurons in thawed cultures

**Fig. 4:**
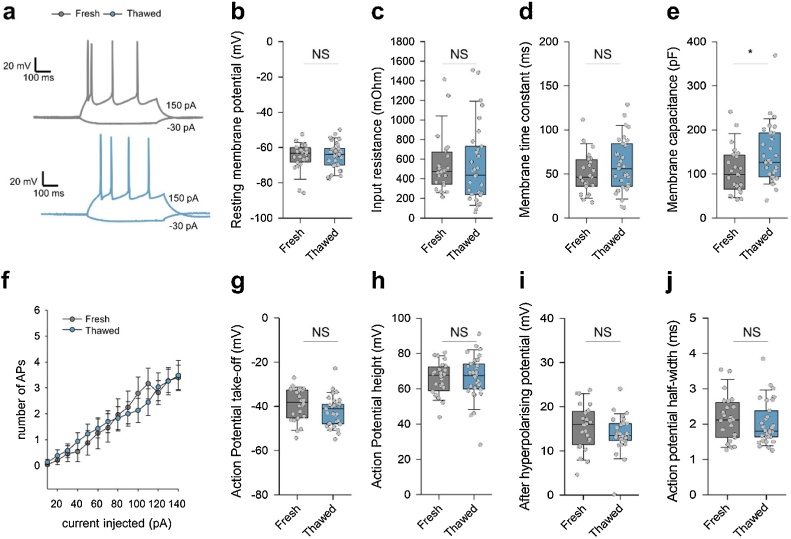
Comparison of neuronal excitability and passive membrane properties in pyramidal neurons in hippocampal cultures from thawed and from freshly dissociated tissue. (**a**) Representative whole-cell membrane potential traces in response to current injection (200 pA, 500 ms) recorded from putative pyramidal neurons (see Methods) from freshly dissociated (top) and from thawed (bottom) cultures at 15 DIV. (**b**-j) Quantification of active and passive neuronal membrane properties: (**b**) resting membrane potential, (**c**) input resistance, (**d**) membrane time constant, (**e**) membrane capacitance, (**e**) neuronal excitability, (**g**) somatic action potential take-off voltage, (**h**) AP amplitude. (**i**) after hyperpolarising potential, (j) AP half-width. We found no statistically significant differences for all the functional parameters tested, except for an approximately 25% higher membrane capacitance in pyramidal neurons from thawed cultures. Data are from n = 24 cells from m = 8 freshly dissociated cultures and n = 31 cells from m = 9 thawed cultures, NS p > 0.2,* p < 0.05, Mann–Whitney U test.

**Fig. 5:**
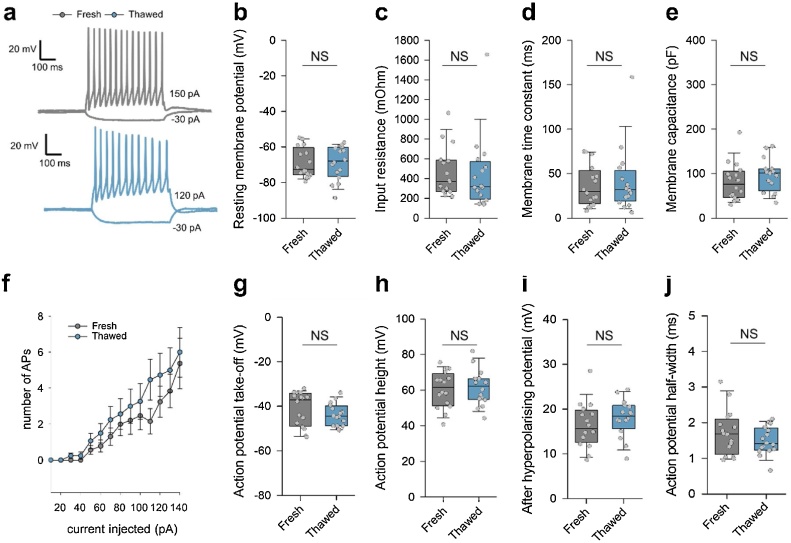
Comparison of neuronal excitability and passive membrane properties in interneurons in hippocampal cultures from thawed and from freshly dissociated tissue. (**a**) Representative whole-cell membrane potential traces in response to current injection (200 pA, 500 ms) recorded from putative interneurons (see Methods) from cultures prepared from freshly dissociated (top) and thawed (bottom) tissue at 15 DIV. (**b**-j) Quantification of active and passive neuronal membrane properties: (**b**) resting membrane potential, (**c**) input resistance, (**d**) membrane time constant, (**e**) membrane capacitance, (**e**) neuronal excitability, (**g**) somatic action potential take-off voltage, (**h**) AP amplitude. (**i**) after hyperpolarising potential, (j) AP half-width. We found no statistically significant differences for all the functional parameters tested. Data are from n = 16 cells from m = 8 freshly dissociated cultures and n = 16 cells from m = 9 thawed cultures, NS p > 0.2,* p < 0.05, Mann–Whitney U test.

We next compared passive and active electrical properties of neurons from cryopreserved/thawed and from fresh hippocampi. Cells were classified into pyramidal neurons or interneurons based both on soma morphology and spiking patterns. Pyramidal neurons had larger somata, often with distinct apical dendrites, and regular low-frequency firing patterns (sometimes with a short initial burst) (Bean, 2007) (Fig. 4); while interneurons had smaller, round cell bodies, with typically fast or irregular spiking patterns, combined with a prominent after-hyperpolarisation (Ascoli et al., 2008) (Fig. 5). Most of the passive and active membrane properties were indistinguishable between freshly dissociated and thawed neurons, including resting membrane potential, input resistance, membrane time constant, number of spikes upon current injection, AP take off voltage, AP amplitude and after-hyperpolarization potential (Fig. 4, Fig. 5). The only significant difference observed was in membrane capacitance, which was approximately 30% larger in neurons prepared from frozen tissue than in neurons prepared from fresh tissue (Fig. 4e). These results indicate that cryopreserved neurons have similar electrical properties to their freshly prepared counterparts.

### 3.4 Comparison of AP-evoked vesicle release in thawed and freshly dissociated neurons

**Fig. 6:**
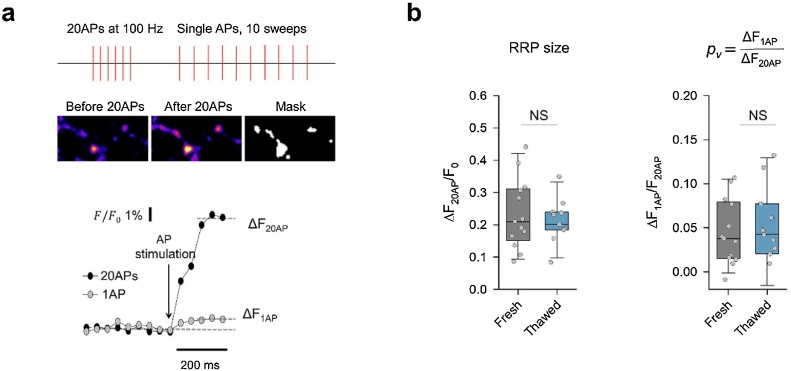
SV release probability and functional SV pool sizes are similar in hippocampal cultures from thawed and freshly dissociated tissue. (**a**) Representative sypHy imaging experiment designed to determine the relative RRP size (ΔF_20APs_) and the average release probability of RRP vesicles p_v_ in response to single APs (p_v_ = ΔF_1AP_ / ΔF_20APs_) (Ariel and Ryan, 2010). Top, experimental paradigm and sypHy fluorescence images before and after a 20 APs 100 Hz burst. The binary ‘Mask’ image identifying active synaptic boutons was obtained by subtracting the resting sypHy fluorescence (Before 20 APs) from the peak fluorescence immediately after the AP burst. Right, corresponding traces of sypHy responses. (**b**) Summary box-and-dot plots showing that RRP size and p_v_ are similar in fresh and thawed neurons. Data are from n = 11 independent imaging experiments from m = 8 freshly dissociated cultures, and from n = 13 experiments from m = 9 thawed cultures, NS p > 0.7, Mann–Whitney *U test*.

Finally, we evaluated functional synaptic properties of thawed neurons. For this purpose, we transduced fresh and thawed neurons at 7 DIV with a lentiviral construct encoding the presynaptic fluorescence reporter synaptophysin-pHluorin (SypHy) (Granseth et al., 2006) and estimated the size of the readily releasable pool (RRP) of vesicles and the average release probability of individual RRP vesicles (*p_v_*). SypHy fluorescence imaging was performed at 15 DIV (Fig. 6). We first recorded the increase in sypHy fluorescence in response to a burst of 20 APs at 100 Hz (ΔF_20APs_). This stimulation protocol was previously shown to trigger release of the entire RRP of vesicles (Ariel and Ryan, 2010). Then we measured the SypHy response (averaged over 10 trials) to single AP stimulation (ΔF_1AP_), and estimated the average release probability of individual RRP vesicles as *p_v_* = ΔF_1AP_/ΔF_20APs_ (Ariel and Ryan, 2010). Presynaptic boutons in cryopreserved and fresh neurons had similar RRP sizes and *p_v_*, thus further arguing that thawed neurons are functionally similar to their freshly prepared counterparts.

## 4 Discussion

Dissociated primary neuronal cultures represent a powerful tool for exploring neuronal physiology. In particular, the use of cultures prepared from mouse knock-out and knock-in models allows one to investigate the specific functions of neuronal proteins in a controlled environment. This however requires an establishment and maintenance of genetically modified mice colonies in the laboratory. To facilitate long-distance collaborations among research groups that use neuronal cultures from genetically modified mice we developed a simple protocol for cryopreservation of mouse hippocampi from new-born pups.

Cryopreservation is a routinely used method in both research and clinical applications to allow long-term storage of mammalian cells and tissues. Indeed, preparation of functional primary cultures from pre-dissociated cryopreserved rodent embryonic cortical neurons has been reported by several studies (Otto et al., 2003; Pischedda et al., 2018; Quasthoff et al., 2015). These preparations however, involve an enzymatic dissociation of neuronal tissue prior to freezing and thus require considerable time and a fully equipped tissue culture facility. We capitalised instead on earlier studies describing cryopreservation of intact neuronal tissue (Kawamoto and Barrett, 1986; Negishi et al., 2002a, b; Rahman et al., 2010), and developed a protocol that allows brain tissue to be frozen directly at the animal facility. Previously, Kawamoto and Barret (Kawamoto and Barrett, 1986) systematically tested physical and biochemical parameters essential for short-term hibernation (at 3–8 °C for up to 7 days) and for long-term storage (between –70 °C to– 90 °C for up to several months) of brain tissue to prepare viable primary neuronal cultures. In our work we further optimised the Kawamoto and Barret protocol using modern commercially available neuronal cultures media and freezing equipment.

In addition to reducing the time of enzymatic digestion, the two other critical steps in the current cryopreservation protocol are controlled gradual freezing (at approximately 1 °C / minute) and storage below −80 °C. When developing this protocol, we found that some cells can indeed survive storage at higher temperatures (*e.g.* −20 °C) but their viability usually decreases, affecting culture quality and reliability. These observations are in line with a previous study (Kawamoto and Barrett, 1986), which reported that storage at –90 °C preserved more than three times as many cells as did storage at −20 °C.

The protocol described here allows one to prepare functional primary hippocampal neuronal cultures from cryopreserved tissue, which can be shipped on dry ice among collaborating laboratories. Importantly, we show that hippocampal neurons in cultures from cryopreserved tissue possess physiological properties similar to those in cultures prepared from freshly dissociated tissue, including neuronal morphology, relative abundance of excitatory and inhibitory neurons and proportion of glial cells, neuronal excitability, AP-waveform and synaptic neurotransmitter release. Among all functional parameters tested we observed only two differences between freshly prepared and thawed cultures: a change in astrocyte shape (Fig. 3) and a 30% increase of membrane capacitance (size) of pyramidal neurons (Fig. 4). These differences could be due to the effect of cryopreservation procedure on the efficiency of neuronal and glial cell attachment to the coverslips. It is however unlikely, that the increase in membrane capacitance and change in glial cell morphology have a direct impact on synaptic release of neurotransmitters. Indeed, we didn’t find any differences in functional presynaptic parameters between the two types of cultures measured at the level of single synapses using the sypHy imaging assay (Fig. 6).

In the present work we specifically focused on preparation of primary neuronal cultures from cryopreserved hippocampi. Although We have not tested this systematically, we also find that the described protocol can be used to prepare primary cultures from cryopreserved neocortical tissue. However, cryopreservation of cortical tissue is likely to require specific optimisation of the freezing (size of the tissue pieces) and the enzymatic digestion steps.

In comparison to the cryopreservation protocols that include enzymatic digestion prior to freezing (Otto et al., 2003; Pischedda et al., 2018; Quasthoff et al., 2015), the main advantage of the protocol described here is its speed. Indeed, it takes less than 30 min to perform dissection and freezing of hippocampi from several mouse pups in parallel (up to 5–7). We thus anticipate that this method will further facilitate collaborations among laboratories based at distant locations and will thus optimise the use of genetically modified mouse models, in line with the 3Rs (Replacement, Reduction and Refinement) recommended for scientific use of animals in research (Prescott and Lidster, 2017).

## Declaration of Competing Interest

The authors declare that they have no competing interests.
